# The complete chloroplast genome of Walla Patta, *Gyrinops walla* (Thymelaeaceae), an agarwood-producing tree species from Sri Lanka

**DOI:** 10.1080/23802359.2021.1926362

**Published:** 2021-05-19

**Authors:** Jingrui Chen, Shiou Yih Lee, K. D. Munugoda, Rozi Mohamed, S. M. C. U. P. Subasinghe, Wenbo Liao

**Affiliations:** a School of Life Science, State Key Laboratory of Biocontrol and Guangdong Provincial Key Laboratory of Plant Resources, Sun Yat-sen University, Guangzhou, China; b Department of Forestry and Environmental Science, Centre for Forestry and Environment, University of Sri Jayewardenepura, Nugegoda, Sri Lanka; c Department of Forestry Science and Biodiversity, Faculty of Forestry and Environment, Forest Biotechnology Laboratory, Universiti Putra Malaysia, UPM Serdang, Seri Kembangan, Malaysia

**Keywords:** CITES, fragrant resin, next-generation sequencing, phylogenomics, traditional medicine

## Abstract

*Gyrinops walla* is an important agarwood-producing tree and threatened species from Sri Lanka. Herein, we assembled and characterized the complete chloroplast (cp) genome of *G. walla* as a genomic resource for conservation purposes. The 175,130 bp long genome is comprised of 87,376 bp large single-copy (LSC) and 3316 bp small single-copy (SSC) regions, which are separated by two inverted repeat (IR) region, each with a size of 42,291 bp. A total of 140 genes were predicted for the cp genome, which includes 94 protein-coding, 38 tRNA, and eight rRNA genes. Phylogenetic analysis showed that *G. walla* is fully resolved in a sister position to *Aquilaria* in the family Thymelaeaceae. The data provided will be useful for study on the molecular phylogenetics and evolution of Thymelaeaceae in the future.

*Gyrinops walla* Gaertn. 1791, locally known as ‘Walla Patta’, is naturally distributed in the low and mid elevations of the wet zone of Sri Lanka (Subasinghe et al. 2012). The species is mainly confined to natural forests and related vegetation, where it is associated with other native species in the lower canopy layer (Subasinghe and Hettiarachchi 2013). The plant is known to produce fragrant resin, called the agarwood. The agarwood is sought after as raw material for perfumery and traditional Attar (Subasinghe and Hettiarachchi 2013). In the wild, the tree is heavily poached and illegal agarwood hunters tend to conduct illegal felling of these trees in search of the agarwood, causing its population to decrease dramatically over time. Therefore, the Sri Lankan Government listed this species under ‘Vulnerable category’ in 2012 and banned transporting and exporting of any part of the tree or product (Subasinghe 2013). To date, *G. walla* is classified under the Appendix II of the Convention on International Trade in Endangered Species (CITES) and all international trade of this species is closely monitored (UNEP-WCMC (Comps) 2021). Scientific studies were widely conducted on *G. walla* after identifying its ability of producing agarwood resins (Subasinghe and Hettiarachchi 2013). However, genetic research on this species is still limited (Eurlings and Gravendeel 2005; Farah et al. 2018; Pern et al. 2020). In this study, we characterized the complete chloroplast (cp) genome sequence of *G. walla* to serve as a valuable genomic resource for the conservation effort of this important agarwood-producing plant species and to determine its evolutionary relationship to other genera classified in the Thymelaeaceae.

Total genomic DNA was extracted using the cetyltrimethylammonium bromide (CTAB) method (Doyle and Doyle 1990) from fresh leaves of wild *G. walla* collected from a forest associated vegetation in the Yagirala of Kalutara District (N06°21′48″ E80°10′08″). A voucher specimen was deposited in the Department of Forestry and Environmental Science, University of Sri Jayewardenepura, Sri Lanka (https://www.sjp.ac.lk; S.M.C.U.P. Subasinghe; upuls@sjp.ac.lk) under the collection number GWYAG01-06. Using the TruSeq DNA Sample Prep Kit (Illumina, San Diego, CA), a 300 bp insert size genomic library was constructed and sequencing was carried out on the Illumina Novaseq platform. Approximately, 2 Gb of 150 bp paired-ends raw data were generated and NOVOPlasty 4.3 (Dierckxsens et al. 2017) was used for the genome assembly. The *rbc*L sequence of *G. walla* (GenBank accession number: MF443411) was designated as the seed sequence. Gene annotation was conducted using GeSeq 2.03 (Tillich et al. 2017) based on default parameters and manually checked for errors.

The complete cp genome sequence of *G. walla* (GenBank accession number: MW557323) exhibited a typical quadripartite structure and has a length of 175,130 bp. The cp genome includes a large single-copy (LSC) region of 87,376 bp, a small single-copy (SSC) region of 3316 bp, separated by a pair of 42,219 bp inverted repeat (IR) regions. A total of 140 genes were predicted, including 94 protein-coding, 38 tRNA, and eight rRNA genes. The overall GC content was 36.7%.

To determine the phylogenetic placement of *G. walla* within the family Thymelaeaceae using the complete cp genome sequences, 12 selected taxa from the family Thymelaeaceae were aligned using MAFFT 7.470 (Katoh and Standley 2013) and phylogenetic analysis was conducted using the maximum-likelihood (ML) method with RAxML (Stamatakis 2014) pipeline available in the CIPRES Science Gateway (Miller et al. 2010). The ML tree was constructed using the general-time reversible (GTR) with gamma distribution (+G) (=GTR + G) nucleotide substitution model, with 1000 bootstrap replicates. Two species, *Gossypium hirsutum* (Malvaceae) and *Eucalyptus grandis* (Myrtaceae) were included as outgroups. The ML tree fully resolved *G. walla* in a clade with three species of *Aquilaria* (Figure 1). The molecular placement of *G. walla* using the complete cp genome sequences appears to be in line with previously reported finding using the intergenic spacer region *trn*L-*trn*F (Eurlings and Gravendeel 2005) and the combined dataset of *mat*K, *rbc*L, *trn*L intron, *trn*L-*trn*F, and *psb*C-*trn*S (Farah et al. 2018).

**Figure 1.**
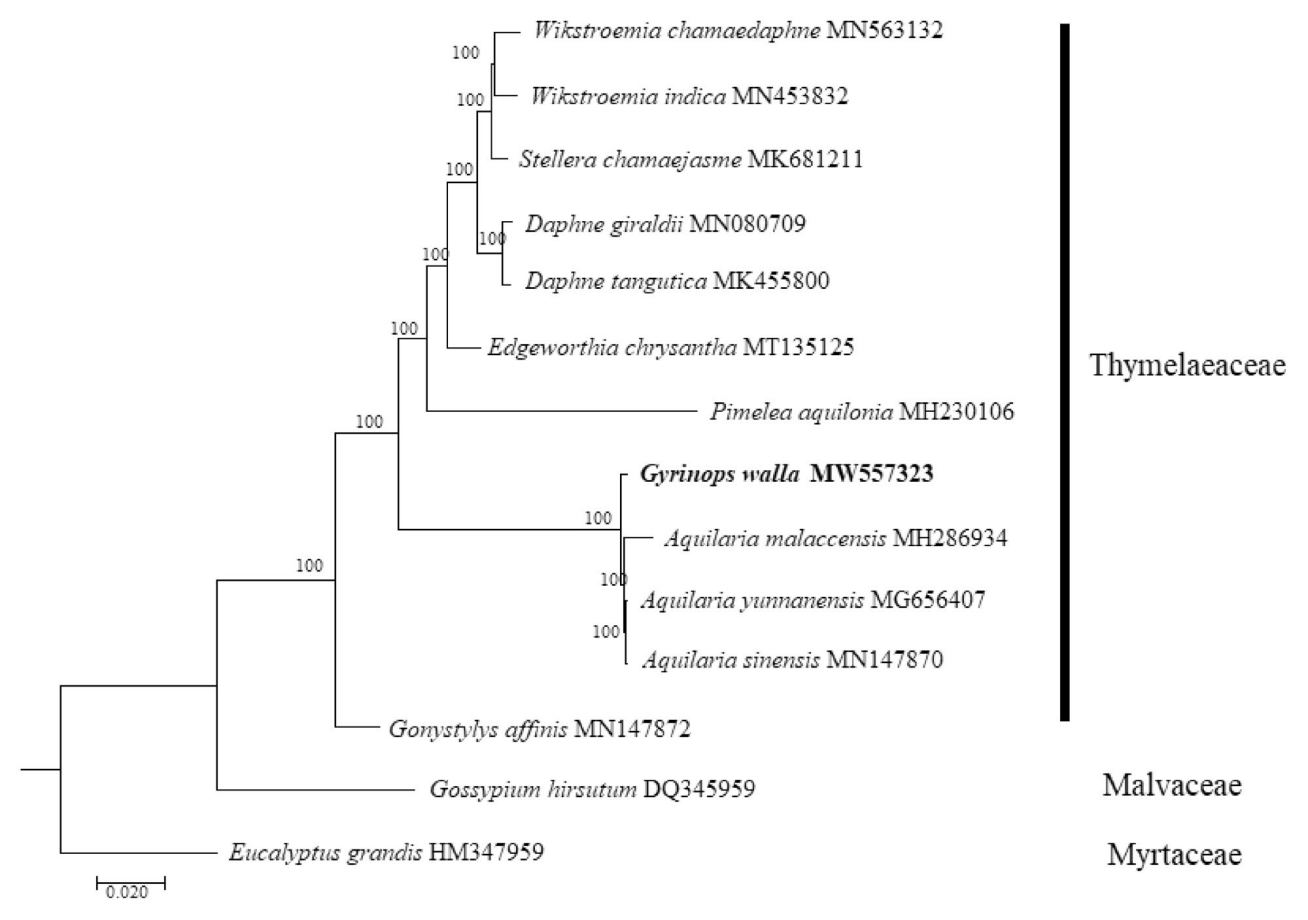
Maximum-likelihood tree constructed based on the complete chloroplast genome sequences of 12 plant taxa from the family Thymelaeaceae, with *Gossypium hirsutum* (Malvaceae) and *Eucalyptus grandis* (Myrtaceae) as outgroups. All branch nodes are indicated with bootstrap support values based on 1000 replicates.

## Data Availability

The genome sequence data that support the findings of this study are openly available in GenBank of NCBI at http://www.ncbi.nlm.nih.gov under the accession number MW557323. The associated BioProject, SRA, and BioSample numbers are PRJNA698718, SRX10001997, and SAMN17734768, respectively.
